# Peroral endoscopic myotomy for complex achalasia and the POEM difficulty score: An update

**DOI:** 10.1002/deo2.70055

**Published:** 2025-01-23

**Authors:** Carmen Ching Hui Yee, Michael Youssef, Matthew Woo, Robert Bechara

**Affiliations:** ^1^ Department of Internal Medicine McGill University Montreal Quebec Canada; ^2^ Department of Internal Medicine University of Toronto Toronto Ontario Canada; ^3^ Division of Gastroenterology Department of Medicine University of Calgary Calgary Alberta Canada; ^4^ Division of Gastroenterology Department of Medicine Queen's University Kingston Ontario Canada

**Keywords:** achalasia, endoscopy, myotomy, natural orifice endoscopic surgery, peroral endoscopic myotomy

## Abstract

**Objectives:**

We present an update on the (peroral endoscopic myotomy (POEM) difficulty score [PDS] by introducing a novel knife with waterjet functionality.

**Methods:**

This is a retrospective review of patients who underwent POEM between May 2018 and July 2023 at the Kingston Health Sciences Center. Demographic and procedural variables were compared using descriptive and inferential statistics.

**Results:**

One hundred thirty‐nine consecutive POEMs were included in the study. Seventy‐four (56.7% male; aged 56.7 ± 16.5 years) complex achalasia (CA) and 65 (55.4% female; aged 47.3 ± 20.2 years) non‐CA POEM procedures were performed. PDS correlates moderately with procedural efficiency with a correlation coefficient of 0.595 (Spearman's *p* < 0.001). The mean efficiency for non‐CA was 3.3 ± 1.2 min/cm compared to CA as follows: type III 3.3 ± 1.3 min/cm; prior myotomy 5.3 ± 2.3 min/cm; ≧4 prior procedures 4.0 ± 1.7 min/cm; sigmoid type 5.2 ± 2.4 min/cm. The median PDS for non‐CA was 1 (1–5). In comparison, the median PDS for CA is as follows: type III 3 (2–4); prior myotomy 4 (3–5); ≧4 prior procedures 3 (1.25–4); sigmoid type 3 (2–4). PDS excluding the presence of spastic contractions correlated better with procedural velocity, with a correlation coefficient of 0.645 (Spearman's *p* < 0.001).

**Conclusions:**

PDS continues to moderately correlate with procedural efficiency using the novel knife. The presence of spastic contractions correlated poorly with procedural efficiency. Thus, it may be omitted in further studies.

## INTRODUCTION

Achalasia is a rare motility disorder characterized by impaired relaxation of the lower esophageal sphincter (LES) and loss of peristaltic contractions in the esophageal body.^1^ Clinical symptoms include dysphagia, regurgitation, chest pain, and weight loss.^2^ Traditional treatment modalities included botulinum toxin (botox) injections, pneumatic dilation (PD), or laparoscopic Heller myotomy (LHM). More recently, Peroral Endoscopic Myotomy (POEM) has emerged as a minimally invasive therapy with high efficacy, even among patients who failed prior endoscopic or surgical therapies.^3^, ^4^, ^5^ However, POEM can be technically challenging in complex achalasia (CA) which includes type III achalasia, sigmoid type, prior myotomy, or multiple prior treatments.^6^ These features are associated with prolonged procedural time, higher failure rates, and perioperative complications.^7^

In 2018, Bechara et al. developed a POEM Difficulty Score (PDS) to describe procedural difficulty based on procedure‐related factors including fibrosis, oozing, orientation, distension of submucosal tunnel, and spastic contractions during the procedure. (Table 1)^6^ Using the traditional triangle tip (TT) knife, the PDS correlated well with procedural velocity and difficulty and may indirectly predict postoperative success and complications.^6^ A limitation is the need to exchange the instrument for an injection needle to expand the submucosa with saline to facilitate dissection.^8^ More recently, the TT‐jet (TTJ) knife with waterjet functionality integrates dissection with an injection to decrease instrument exchanges during POEM.^8^ Consequently, it was shown to decrease operative time and increase procedural efficiency, regardless of PDS score.^8^

**TABLE 1:** Peroral endoscopic myotomy difficulty score fibrosis, oozing, orientation, distention of tunnel, and spastic contractions variables.

FOODS variables	Scoring system
Fibrosis	a. None to minimal b. Moderate c. Severe
Oozing	a. None to minimal b. Moderate c. Diffuse
Orientation	a. Easy b. Fair c. Difficult
Distension of tunnel	a. Excellent b. Adequate c. Poor
Spastic contractions	a. Seldom or none b. Moderately frequent c. Very frequent

In this case series, we aim to report on the clinical and procedural efficacy of using the TTJ knife in POEM for a larger series of patients with CA and non‐CA. Additionally, we explore and update the correlation between PDS and procedural variables to better describe procedural difficulty in CA.

## METHODS

### Study design

#### PDS

The PDS describes procedural variables that impact the technical difficulty of performing POEM.^9^, ^10^, ^11^ This score consists of five variables: fibrosis, oozing, orientation, distention of tunnel, and spastic contractions.^6^ Each variable is weighted equally, and the values assigned ranged from 0 to 2. (Table 1) Details regarding each variable and its scoring system have been previously described.^6^

#### Fibrosis

It is graded as follows: 0, minimal to no fibrosis (submucosa appears as a blue transparent layer); 1, mild‐moderate fibrosis (submucosa appears as a white web‐like tissue); 2, severe fibrosis (submucosa appears as a white muscle‐like structure without a blue transparent layer).^6^, ^14^

#### Orientation

It is graded as follows: 0, easy (no difficulty with orientation, tunnel easily advanced perpendicular to the circular muscle without significant reorientation required); 1, fair (slightly challenging orientation; however, easily reoriented with repeated withdrawal and advancement of the scope); 2, difficult (despite repeated withdrawal and advancement, orientation remains challenging).^6^

#### Oozing

It is graded as follows: 0, absent/minimal submucosal oozing (non‐pulsatile bleeding, easily identified and treated without subsequent impairment in delineation of tissue planes); 1, moderate oozing (non‐pulsatile bleeding identified and controlled, may have a transient impact on subsequent delineation of tissue planes); 2, diffuse oozing (dispersed oozing that is difficult to identify a clear source and results in impaired visibility/ability to delineate tissue planes).^6^

#### Distension of tunnel

It is graded as follows: 0, excellent (minimal to intermittent requirement for insufflation to maintain optimal working space); 1, adequate (persistent insufflation required to maintain optimal working space); 2, poor (even with continuous insufflation, the optimal working space cannot be achieved).^6^

#### Spastic contractions

This refers to the frequency of abnormal spastic contractions encountered during the procedure. These contractions are felt and seen by the operator and result in impaired visualization and maneuverability. It is graded as follows: 0, seldom or no spastic contractions; 1, spastic contractions every 6–10 s; 2, spastic contractions occurring every 0–5 s.^6^

### Patient characteristics

Patients classified as CA have either type III achalasia, prior myotomy, four or more previous endoscopic treatments (only botox injection or PD 30  mm or more were counted; multiple bougie and/or balloon dilations <30  mm or dilations for which details were unknown were not sufficient to be classified as CA), and sigmoid type.^6^ The primary outcome examined was clinical success defined by an Eckardt score of 3 or less at a minimum of 3 months follow‐up with the use of TTJ knife. Secondary outcomes examined include the correlation of PDS with procedural velocity in the CA subtype, further stratification of features associated with CA, and the correlation of PDS with preprocedural patient characteristics and symptoms. Procedural velocity was defined as total procedure time (min) divided by total myotomy length (cm). Procedural velocity will be interchangeably used with the term procedural efficiency.

Clinical, procedural, and demographic data were collected prospectively and retrospectively analyzed. A total of 139 consecutive patients undergoing POEM at a single tertiary care center between May 2018 and July 2023 were included in this series. The sole operator for this study (Robert Bechara) had undergone a formal 1‐year fellowship at Showa University, Digestive Diseases Center, under tutelage from Haruhino Inoue, the pioneer of POEM.^15^ Diagnosis of achalasia was made by high‐resolution esophageal manometry using the Chicago classification 4.0.^16^

### Data collection

Clinical data collected for this study included age, gender, body mass index, American Society of Anesthesiologists (ASA) score, types of symptoms, duration of symptoms in months, achalasia subtype, prior treatment for achalasia, pre‐procedural Eckardt scores and integrated relaxation pressures (IRP). Procedural data included the length of myotomy, number of clips used for closure, operative time, PDS, intra/post‐procedural complications, and length of hospital stay. Complications were classified according to the Clavien‐Dindo classification.^17^ Post POEM follow‐up data include Eckardt scores, number of months since the procedure, follow‐up upper endoscopy findings, 24‐h pH testing off proton‐pump inhibitor (PPI) therapy (ZAN‐BG‐44; Diversatek Healthcare), and manometry (HRiM2 12 Fr Probe; Diversatek Healthcare) for follow‐up IRP.

The pre‐ and post‐POEM protocol used during this study remained unchanged from the prior.^6^ There were no changes in the techniques used by the operator (Robert Bechara). There were no changes to the definition of the PDS from the previous study.^6^ All cases in this cohort were completed using a TTJ knife.

### Data analysis

Demographics and procedural variables were analyzed with descriptive statistics (mean ± standard deviation {SD}; median, interquartile range [IQR]) and the Mann‐Whitney U‐test. The Eckardt scores pre‐ and post‐POEM were compared using the Wilcoxon signed‐rank test. Spearman's correlation (Significance defined as *p* < 0.05) was used to analyze the relationship between PDS and procedural velocity. The correlation between preprocedural variables, symptoms, and PDS was analyzed with Spearman's correlation and Kruskal Wallis H test (Significance defined as *p* < 0.05). All calculations were performed using SPSS v29.

## RESULTS

One hundred thirty‐nine consecutive POEMs were performed, with seventy‐four being CA. Demographic characteristics of patients with CA and non‐CA are shown in Table 2. Patients with CA had a significantly longer duration of symptoms (median 93 months, IQR 24–145.5) compared to non‐CA (median 36 months, IQR 19.5–96) (*p* = 0.002). Patients with CA have higher mean body mass index (27 [SD 6.1] vs. 23.3 [SD 4.2]) (*p* < 0.001) and were slightly older than the non‐CA group (mean age 56.7 [SD 16.5] vs. 47.3 [SD 20.2]) (*p* = 0.009). The ASA score was greater in CA (median 3, IQR 2–3) compared to non‐CA (median 2, IQR 2–3) (*p* = 0.013). Pre‐Eckardt scores were not significantly different between the groups (*p* = 0.076).

**TABLE 2:** Demographic characteristics of patients with complex achalasia and non‐complex achalasia.

Baseline characteristics	Complex	Non‐complex	*p‐*value
Total patients (male:female)	74 (42:32)	65 (36:29)	
Age (years) [mean, SD]	56.7 ± 16.5	47.3 ± 20.2	0.009
BMI (kg/m2) [mean, SD]	27.0 ± 6.1	23.3 ± 4.2	<0.001
ASA class (median, IQR)	3 (2–3)	2 (2–3)	0.013
Duration of symptoms in months (median, IQR)	93 (23–145.5)	36 (19.5–96)	0.002
Achalasia subtype (total)	74	65	
Type I	18	7	
Type II	15	54	
Type III	39	N/A	
Unclassified	2	4	
Sigmoid (total)	34		
S1	28		
S2	6		
4 or more previous treatments (total)	8		
Median number of treatments (*n*, range)	0 (0–20)		
Pneumatic dilation	12		
Bougie or dilation not specified	14		
Botulinum toxin injection	16		
Prior myotomy	15		

IQR, interquartile range; BMI, body mass index defined as weight in kilograms (kg) divided by height (m^2^); ASA, American Society of Anesthesiologists.

Of those classified as CA, it was comprised of type III (*n* = 39), sigmoid type (*n* = 34), and 4 or more previous treatments (*n* = 8). Many CA patients had prior treatments, notably PD (*n* = 12), bougie or dilation (*n* = 14), botox injection (*n* = 16), or prior myotomy (*n* = 15).

The preprocedural outcomes are available in Table 3. Patients with CA have significantly greater PDS (median 3, IQR 2–4) compared to non‐CA (median 1, IQR 1–2; *p* < 0.001). This corresponds to a longer operative time (mean 52.1 min [SD 20.4] vs. 42.1 min [SD 14.9]; *p* = 0.003). The mean procedural velocity (min/cm myotomy) was significantly greater in CA (4.2 [SD 2.2] vs. 3.3 [SD 1.2]; *p* = 0.008).

**TABLE 3:** Peroral endoscopic myotomy procedural outcomes.

Procedural results of patients undergoing POEM	Complex	Non‐complex	*p*‐value
Total	74	65	
Myotomy length (cm) [mean, SD]	13.6 ± 4.8	13.4 ± 4.2	0.800
Esophageal myotomy length (cm) [mean, SD]	11.6 ± 4.9	11.4 ± 4.1	0.851
Gastric myotomy length (cm) [mean, SD]	1.9 ± 0.4	1.9 ± 0.4	0.851
Clip (number) [median, IQR]	4 (4–5)	4 (4–5)	0.264
Operative time (min) [mean, SD]	52.1 ± 20.4	42.1 ± 14.9	0.003
PDS (median, IQR)	3 (2–4)	1 (1–2)	<0.001
Fibrosis (median, IQR)	1 (0–1)	0 (0–1)	
Oozing (median, IQR)	0 (0–0)	0 (0–0)	
Ease of orientation (median, IQR)	1 (0–1)	0 (0–1)	
Distension of tunnel (median, IQR)	1 (1–1)	1 (0–1)	
Presence of spastic contractions (median, IQR)	0 (0–1)	0 (0–0)	
Velocity (min/cm myotomy) [mean, SD]	4.2 ± 2.2	3.3 ± 1.2	0.008
Length of stay (days) [median, IQR]	1 (1‐1)	1 (1‐1)	0.892
Major complications (Clavien‐Dindo grade IIIb‐V)	0	1*	

IQR, interquartile range; PDS, peroral endoscopic myotomy difficulty score; POEM, peroral endoscopic myotomy.

*1 Clavien‐Dindo grade IIIb event – mucosotomy requiring clip ×3.

There were no differences in the total myotomy length or length of stay between CA and non‐CA patients. The non‐CA cohort had a grade I and IIIb complication, respectively. There was a leak because of clips falling off 3 days post‐discharge, managed conservatively, and a mucosotomy requiring an additional three clips without any clinical consequences.

The PDS correlated moderately with procedural velocity with a correlation coefficient of 0.595 (Spearman's *p* < 0.001). The procedural velocity for non‐CA was 3.3 min/cm [SD 1.2]. In comparison, procedural velocity for CA was 4.2 min/cm (SD 2.2; all CA types), 3.3 min/cm (SD 1.3; type III), 5.3 min/cm (SD 2.3; prior myotomy), 4.0 min/cm (SD 1.7; ≥4 prior procedures), and 5.2 min/cm (SD 2.4; sigmoid type). The procedural velocity of CA excluding type III achalasia was 4.9 min/cm (SD 2.4). A separate analysis of individual PDS factors demonstrated a poor correlation between the presence of spastic contractions and procedural velocity at 0.1 (Spearman's *p* = 0.906). Meanwhile, the correlation coefficient between fibrosis and efficiency was 0.520 (Spearman's *p* < 0.001); Oozing and efficiency 0.323 (*p* < 0.001); Ease of orientation and efficiency 0.351 (*p* < 0.001); Distension of tunnel and efficiency 0.4 (*p* < 0.001). PDS not including the presence of spastic contractions correlated better with the procedural velocity at 0.645 (Spearman's *p* < 0.001).

The PDS correlated weakly with preprocedural variables; ASA (Spearman's 0.269, *p* = 0.002); body mass index (Spearman's 0.256, *p* = 0.002); sex (*p* = 0.471); age (Spearman's 0.219, *p* = 0.001); pre‐Eckardt score (Spearman's = 0.094, *p* = 0.271), and symptoms (dysphagia, *p* = 0.198; regurgitation, *p* = 0.252; chest pain, *p* = 0.28; weight loss, *p* = 0.447). The PDS correlated moderately with duration of symptoms (Spearman's 0.344, *p* < 0.001). We were unable to identify any demographic or clinical variables that strongly correlate with difficulty.

Table 4 describes the post‐POEM follow‐up period. The median follow‐up period was 27 months (IQR 17–35) in CA and 21.5 months (IQR 11.25–40.75) in non‐CA. There was a significant improvement in Eckardt scores post‐procedure with 97–98% of patients with post‐POEM Eckardt scores of 3 or less.

**TABLE 4:** Post‐peroral endoscopic myotomy outcomes.

Follow up EGD	Complex	Non‐complex	*p*‐value
Total (*n*, %)	64 (86.5%)	52 (80%)	
Esophagitis (*n*, %)	26 (40.6%)	25 (48%)	0.671
LA A (*n*)	7	7	
LA B (*n*)	13	16	
LA C (*n*)	6	2	
Follow up 24 h pH study total (n, %)	19 (25.7%)	21 (32.3%)	
Abnormal pH < 4 (*n*)	12	10	0.071

EGD, esophagogastroduodenoscopy.

Follow‐up endoscopy was completed 3–6 months post‐POEM in 64 (86.5%) CA and 52 (80%) non‐CA patients. Esophagitis was classified according to the LA classification.^18^ Esophagitis was found in 26 (40.6%; LA A, seven patients; LA B, 13 patients; LA C, six patients) and 25 (48%; LA A, seven patients; LA B, 16 patients; LA C, two patients) CA and non‐CA patients, respectively. Abnormal pH exposure was defined as esophageal pH < 4 greater than 4.2% of total time. This was seen in 63.1% and 47.6% of CA and non‐CA patients, respectively. Based on the Lyon 2.0 consensus, 29.7% and 34.6% of CA and non‐CA patients met the criteria for gastroesophageal reflux disease (GERD).^19^

## DISCUSSION

In this case series, we present data further supporting the efficacy and safety of POEM for patients with both CA and non‐CA. Furthermore, we demonstrate improvement in procedural velocity, especially in CA with the introduction of the TTJ knife. In addition, we continue to show that PDS correlated with procedural efficiency using the TTJ knife.

### TTJ knife in POEM

There is an increase in the mean procedural velocity between the current and previous cohorts (Tables S1–3). This can be explained using the new TTJ knife in this cohort. Compared to the conventional TT knife, the built‐in injection needle function reduces the mean number of instrument changes by 90%, and the usage of coagulation forceps for hemostasis by 59%.^8^ Tang et al. reported that the conventional TT knife was associated with longer procedural times compared to a hybrid knife with similar functionality as the TTJ knife (*p* = 0.014).^20^

Additionally, there is a reduction in the mean operative time (min) between the current and previous study (Tables S1–3). This is despite a slight increase in the median PDS between the current and previous cohorts.^21^ Although the comparison may be limited due to differences in sample size and improved operator expertise over the study's duration, we are reassured that the threshold for proficiency (up to 60 cases) was well surpassed by the primary operator prior to this cohort's initiation. This is further supported by the similar clinical outcomes observed in both groups and the number of procedures performed by the operator by the time the study was conducted. (Table S4).^22^

### PDS correlation

In this study, we demonstrated that PDS correlates with procedural efficiency with the TTJ knife. There appears to be a reduction in variability in the PDS and procedural efficiency between CA and non‐CA compared to the previous study (Tables S5 and S6).^6^ The correlation coefficient between PDS and procedural efficiency was 0.772 (Spearman's *p* < 0.001) compared to the current value of 0.595 (Spearman's *p* < 0.001). Of note, we observed a poor correlation between the presence of spastic contractions and procedural efficiency possibly due to the ability of the TTJ knife to perform repeated submucosal injections to keep the submucosa well‐lifted, minimizing any effect of spastic contractions.^23^ As such, with an experienced operator and the use of the TTJ knife, type III achalasia can be reclassified as non‐CA, and spastic contractions may be omitted from the PDS.

We observed only a weak correlation between PDS and preprocedural variables (i.e., pre‐procedural Eckardt scores and symptomatology). This is a limitation of PDS due to the inability to use preprocedural variables to predict procedural difficulty pre‐operatively. Future research is needed to investigate other preprocedural variables that might correlate better with the PDS and be used to predict preoperative procedural difficulty.

In comparing the PDS with other scoring systems, Nakai et al. published a risk‐scoring system identifying challenging POEMs.^24^ The scoring system involved a point‐based system with preoperative factors such as duration of symptoms, antithrombotic use, Chicago classification, and grade 3 dilation. These preoperative factors were shown to correlate well with procedural difficulty and complication rates. Expert endoscopists can enhance patient selection for the POEM procedure by utilizing a combined scoring system, including risk assessment and PDS, to predict procedural difficulty and efficiency.^24^, ^25^, ^26^ The PDS could be utilized to track trainee progress by monitoring how variability in procedural efficiency decreases as competency improves. For instance, trainees could be expected to achieve a target efficiency – based on metrics established by the supervisor – without complications for cases with a PDS of 0–2 before progressing to more challenging cases. Additionally, it can serve as a quality assurance tool, allowing POEM operators to record and track outcomes across cases with varying PDS scores.

### CA and difficult POEMs

Tan et al. defined difficult POEMs as POEM with (1) prolonged procedure time, (2) occurrence of major intraoperative adverse events, or (3) aborted procedures owing to the morphological or pathophysiologic changes of the esophagus. Certain characteristics such as sigmoid‐type, submucosal fibrosis, prior treatments, spastic type (type III achalasia, distal esophageal spasm, and hypercontractile esophagus), and achalasia with diverticulum or situs inversus are associated with difficult POEMs.^27^

In our study, type III achalasia had a similar procedural velocity to non‐CA. We also found that spastic contractions correlated poorly with procedural velocity and the PDS. Hence, type III achalasia may not be associated with difficult POEMs.^27^

With respect to postoperative outcomes, both CA and non‐CA patients reported significant improvements in their Eckardt scores following POEM. Importantly, rates of esophagitis and GERD were also similar between the two groups. Previous studies have demonstrated higher rates of reflux esophagitis post‐POEM with alcohol use, obesity, and length of full‐thickness myotomy.^28^ However, the type of achalasia and sigmoid esophagus were not statistically significant, and prior PD or laparoscopic Heller myotomy were in fact protective against GERD.^28^ This suggests that GERD rates may not increase with factors typically associated with CA.

### Limitations and methodological considerations

A notable limitation is the study design. As a single‐center, single‐operator series, generalizability to other populations is limited. Other studies assessing POEM difficulty reported similar methodological limitations.^20^, ^24^, ^26^ Despite a slightly larger number of patients in this series, the number of complications was insufficient to attempt an analysis to identify a correlation with PDS.

## CONCLUSION

In this update to the PDS, it was found that the PDS continues to correlate well with procedural velocity in CA and non‐CA. However, with the introduction of the TT‐J knife, type III achalasia and spastic contractions no longer correlate strongly with procedural velocity or PDS and will be removed from the classification of CA. The PDS can be used to compare cases across different sites, assess POEM difficulty during procedures, and identify instances where novice trainees may benefit from expert intervention. Additionally, the PDS could serve as an adjunct for quality improvement and assurance by tracking outcomes for various types of CA and non‐CA cases across PDS scores. This approach can help identify outliers that indicate the need for quality initiatives or demonstrate that standards are being met for designation as an “expert POEM center”. Future research should involve a more diverse group of operators and patients from multiple centers to validate these findings further.

## CONFLICT OF INTEREST STATEMENT

Robert Bechara: Consulting fees – Vantage Endoscopy, Olympus, Pentax; Payment or honoraria for lectures given – Pendopharm, Pentax, Olympus. All other authors declare no conflict of interest.

## ETHICS STATEMENT

Approval of the research protocol by an Institutional Reviewer Board: N/A

## PATIENT CONSENT STATEMENT

N/A

## CLINICAL TRIAL REGISTRATION

N/A

## ANIMAL STUDIES

N/A

## Supporting information



DENOP_Supplement_2024‐11‐25.docx.
