# Supplementary material for: Peroral endoscopic myotomy for complex achalasia and the POEM difficulty score: An update
Source: DEN Open. 2025 Jan 23;5(1):e70055. doi: 10.1002/deo2.70055 (PMC11757024; doi:10.1002/deo2.70055)
Supplement: Supplementary file 1 — DENOP_Supplement_2024‐11‐25.docx. [file DEO2-5-e70055-s001.docx]

**Supporting Table 1: Procedural outcomes for CA comparing TTJ (Current cohort) and conventional TT knife**

| Procedural results of patients undergoing POEM | Conventional TT | TTJ | *P*-value |
| --- | --- | --- | --- |
| Total | 31 | 74 |  |
| Myotomy length (cm) [mean, SD] | 17.0 ± 2.6 | 13.6 ± 4.8 | <0.001 |
| Esophageal myotomy length (cm) [mean, SD] | 14.6 ± 2.7 | 11.6 ± 4.9 | 0.002 |
| Gastric myotomy length (cm) [mean, SD] | 2.5 ± 0.8 | 1.9 ± 0.4 | <0.001 |
| Clip (number) [median, IQR] | 5 (5-5) | 4 (4-5) | NS |
| Operative time (min) [mean, SD] | 93.3 ± 32.6 | 52.1 ± 20.4 | <0.001 |
| PDS (median, IQR) | 2 (1-4) | 3 (2-4) | 0.029 |
| Fibrosis (median, IQR) | 1 (0-1) | 1 (0-1) | NS |
| Oozing (median, IQR) | 0 (0-0) | 0 (0-0) | NS |
| Ease of orientation (median, IQR) | 0 (0-1) | 1 (0-1) | NS |
| Distension of tunnel (median, IQR) | 1 (0-1) | 1 (1-1) | NS |
| Presence of spastic contractions (median, IQR) | 0 (0-0) | 0 (0-1) | NS |
| Velocity (min/cm myotomy) [mean, IQR] | 5.7 ± 2.5 | 4.2 ± 2.2 | 0.005 |
| Length of stay (days) [median, IQR] | 1 (1-1) | 1 (1-1) | 0.002 |
| Major complications (Clavien-Dindo grade IIIb-V) | 0 | 0 |  |

IQR: Interquartile range
NS: Non-significant

**Supporting Table 2: Procedural outcomes for non-CA comparing TTJ (Current cohort) and conventional TT knife**

| Procedural results of patients undergoing POEM | Conventional TT | TTJ | *P*-value |
| --- | --- | --- | --- |
| Total | 16 | 65 |  |
| Myotomy length (cm) [mean, SD] | 16.1 ± 3.8 | 13.4 ± 4.2 | 0.18 |
| Esophageal myotomy length (cm) [mean, SD] | 13.0 ± 4.4 | 11.4 ± 4.1 | 0.184 |
| Gastric myotomy length (cm) [mean, SD] | 3.1 ± 1.2 | 1.9 ± 0.4 | <0.001 |
| Clip (number) [median, IQR] | 5 (4-5) | 4 (4-5) | NS |
| Operative time (min) [mean, SD] | 74.1 ± 20.2 | 42.1 ± 14.9 | <0.001 |
| PDS (median, IQR) | 1.5 (1-2) | 1 (1-2) | NS |
| Fibrosis (median, IQR) | 0 (0-0) | 0 (0-1) | NS |
| Oozing (median, IQR) | 0 (0-0) | 0 (0-0) | NS |
| Ease of orientation (median, IQR) | 0 (0-0) | 0 (0-1) | NS |
| Distension of tunnel (median, IQR) | 1 (1-1) | 1 (0-1) | NS |
| Presence of spastic contractions (median, IQR) | 0 (0-0) | 0 (0-0) | NS |
| Velocity (min/cm myotomy) [mean, IQR] | 4.8 ± 1.7 | 3.3 ± 1.2 | <0.001 |
| Length of stay (days) [median, IQR] | 1 (1-1) | 1 (1-1) | NS |
| Major complications (Clavien-Dindo grade IIIb-V) | 0 | 1* |  |

IQR: Interquartile range
NS: Non-significant

**Supporting Table 3: Procedural outcomes for all POEMs comparing TTJ (Current cohort) and conventional TT knife**

| Procedural results of patients undergoing POEM | Conventional TT | TTJ | *P*-value |
| --- | --- | --- | --- |
| Total | 47 | 139 |  |
| Myotomy length (cm) [mean, SD] | 16.7 ± 3.1 | 13.5 ± 4.5 | <0.001 |
| Esophageal myotomy length (cm) [mean, SD] | 14.0 ± 3.4 | 11.5 ± 4.5 | <0.001 |
| Gastric myotomy length (cm) [mean, SD] | 2.7 ± 1.0 | 1.9 ± 0.4 | <0.001 |
| Clip (number) [median, IQR] | 5 (4-5) | 4 (4-5) | NS |
| Operative time (min) [mean, SD] | 86.7 ± 30.2 | 47.4 ± 18.7 | <0.001 |
| PDS (median, IQR) | 1.5 (1-2) | 2 (1-3) | NS |
| Fibrosis (median, IQR) | 0 (0-0) | 0 (0-1) | NS |
| Oozing (median, IQR) | 0 (0-0) | 0 (0-0) | NS |
| Ease of orientation (median, IQR) | 0 (0-0) | 0 (0-1) | NS |
| Distension of tunnel (median, IQR) | 1 (1-1) | 1 (0-1) | NS |
| Presence of spastic contractions (median, IQR) | 0 (0-0) | 0 (0-0) | NS |
| Velocity (min/cm myotomy) [mean, IQR] | 5.4 ± 2.3 | 3.8 ± 1.8 | <0.001 |
| Length of stay (days) [median, IQR] | 1 (1-1) | 1 (1-1) | 0.001 |
| Major complications (Clavien-Dindo grade IIIb-V) |  |  |  |

IQR: Interquartile range
NS: Non-significant

**Supporting Table 4: Post-procedure Eckardt score**

|  | Conventional TT (n=47) | TTJ (n=139) |
| --- | --- | --- |
| Eckardt 3 or less (n, %) | 45/47, 95.7% | 116/119*, 97.5% |
| *P*-value | NS | |

NS = 0.622 (Fisher’s Exact Test)
*Missing values – 20

**Supporting Table 5: PDS Correlation coefficient scores with Procedural Velocity**

| PDS Variables | Correlation coefficient | *P*-value |
| --- | --- | --- |
| All PDS variables (Current cohort – TTJ knife) | 0.595 | <0.001 |
| Fibrosis | 0.520 | <0.001 |
| Oozing | 0.323 | <0.001 |
| Orientation | 0.351 | <0.001 |
| Distension of tunnel | 0.400 | <0.001 |
| Spastic contractions | 0.100 | 0.906 |
| PDS excluding spastic contractions | 0.645 | <0.001 |
| PDS excluding spastic contractions and oozing | 0.617 | <0.001 |

**Supporting Table 6: PDS Correlation coefficient scores with Procedural Velocity (Conventional TT)**

| PDS Variables | Correlation coefficient | *P*-value |
| --- | --- | --- |
| All PDS variables | 0.763 | <0.001 |
| Fibrosis | 0.575 | <0.001 |
| Oozing | 0.439 | 0.002 |
| Orientation | 0.532 | <0.001 |
| Distension of tunnel | 0.320 | 0.028 |
| Spastic contractions | 0.098 | 0.510 |
| PDS excluding spastic contractions | 0.782 | <0.001 |
| PDS excluding spastic contractions and oozing | 0.780 | <0.001 |
